# Aspects of Design Thinking, Material and Usability Engineering in the Design of Suit for Police Officers Operating on Motorcycles—Part I: Design and Safety Aspects

**DOI:** 10.3390/ma18174156

**Published:** 2025-09-04

**Authors:** Marcin Henryk Struszczyk, Małgorzata Kudlińska, Tomasz Miedzianowski, Marzena Fejdyś, Agnieszka Gutowska, Katarzyna Kośla, Piotr Suchocki

**Affiliations:** 1 Institute of Security Technologies “MORATEX”, 3 M. Sklodowskiej-Curie Str., 90-505 Lodz, Poland; mkudlinska@moratex.eu (M.K.); tmiedzianowski@moratex.eu (T.M.); mfejdys@moratex.eu (M.F.); agutowska@moratex.eu (A.G.); 2 Institute of Fashion, Władysław Strzemiński Academy of Fine Arts, 121 Wojska Polskiego Str., 91-726 Lodz, Poland; piotrsuchocki@interia.pl

**Keywords:** motorcycle suit, material engineering, design thinking, safety verification, chemical safety

## Abstract

Motorcycle clothing, due to the specific aspects related to its field of use, especially in the area of public safety, requires a special approach already at the design level. The aim of the interdisciplinary research was to develop textile motorcycle suits (variants for summer and winter use) for police officers conducting operational activities on motorcycles. As part of the design research, the principles of design thinking were taken into account, considering material aspects and usability engineering, which allowed for the implementation of a design, material, and usability strategy, as well as the communication message. The developed prototypes of the suits met the requirements of PN-EN 13595 normative documents, indicating that the required safety requirements were met. The introduced principles of design thinking also took into account the aspects of designing a communication message, taking into account the principles of usability technology.

## 1. Introduction

The most accurate way to design special-purpose clothing in the area of design thinking is the hybrid design methodology, as this method of proceeding is called by its experts and promoters, McCann and Bryson [1]. This process emphasizes the recognition of market needs—with particular emphasis on the needs of the “end user” and the adaptation of materials, design features, and technologies to the individual user profile. Direct contact of clothing with the user, especially those carrying out activities in areas requiring special qualifications and at risk of excessive risk, requires entering data related to anthropometric, biomechanical, and ergonomic features as well as non-verbal messages [2,3].

Utility engineering, derived from the design of digital interfaces and IT areas, is also used in the textile and clothing industry. The design of clothing products, especially those used in special areas of dual-use character, defined per user, requires comfort, functionality, intuitiveness of use, and aesthetics. The above-mentioned factors directly affect the satisfaction and experience of the person using the textile-originated product, ensuring the required safety. The current approach to clothing design draws inspiration from research procedures and tools used in usability engineering, such as user needs analysis, prototyping, and multi-threaded validation in real-world conditions, including ergonomics and comfort assessment. In the process of multi-threaded analysis, special emphasis is placed not only on aesthetic aspects (these are important when used in special areas, including in the area of state security and also in the context of subliminal communication messages), but above all on the performance, ease of use, and comprehensibility of the way the product is used. It is important to note that any irregularities in the design can significantly affect the comfort or safety of the user [2,3,4,5].

In the context of the clothing design process, the implementation of digital technologies such as computer simulations, 3D scanning of the body silhouette, avatarization, or virtual fitting also plays an increasingly important role in the context of clothing design to facilitate personalization and assessment of comfort and fit of the product at the initial design stage [6,7,8,9]. Usability engineering is becoming an inseparable element of modern clothing design, translating not only into an increase in the quality of the textile products themselves, but also in the satisfaction and safety of users.

An extremely important aspect in the design of protective products is the definition of risk management measures in the area of operation of the designed product, shaping knowledge about the weight and possible effects of the emerging risks. Motorcyclists are the group of road users with the highest risk of loss of health and life, due to the types of protection they use and the risks resulting from collisions, abrasions, and injuries to the body with sharp objects (glass, sharp stones, vehicle elements, etc.). Statistically, the most common injuries in this group are soft tissue injuries, which can be prevented by using appropriate protective barriers in the form of clothing or suits protecting the most sensitive areas of the body in relation to the assumed injury and zonally protecting the areas of the body that are most often damaged [10,11,12].

In validation tests of motorcyclists’ suits for conditions close to real ones, the standards of the PN-EN 13595 [13] series have been used for a long time, the methodology of which tries to verify the risks resulting from threatening events that occur during road traffic. It should be noted that the data concentrating the results of the tests in accordance with the above-mentioned normative document, due to the long period of its application, allow for a reliable translation of the test results into risks resulting from the occurrence of threatening situations.

According to [8], the probability of soft tissue damage when using motorcycle clothing with a defined Level 1 (in accordance with [13]) ranged from 40% to 60%. Moreover, increasing the abrasion time leads to a reduced risk of soft tissue damage. These studies also confirmed that over 60% of the tested clothing did not meet the minimum requirements of the above-mentioned standard.

Meredith et al. [14] carried out validation studies in relation to the assumptions and requirements of [13] and to actual incidents in road traffic with the participation of the motorcyclists. These studies indicated the correctness of the requirements defined in the above-mentioned normative and the research methodologies defined in parts 2–4 of this standard in relation to real events and zonal exposure of the motorcyclist participating in such events. Quantitative studies have also been conducted to determine the relationship between the use of motorcycle clothing and injuries sustained in accidents [15]. The conclusion of this study was that the use of protective clothing for motorcyclists results in a significant reduction in the risk and severity of injuries associated with an accident and subsequent hospitalization. Another study [16] confirmed that the distribution of impacts and injuries of users resulting from a motorcycle accident is comparable to the four risk zones and the protective effectiveness specified in [13]. The distribution of injuries and their severity corresponded directly to the areas of the zones defined in [13].

The research [17] indicates that it is important to have validated test methodologies and research procedures to verify the safety of motorcyclists’ clothing so that the obtained test results allow for the assessment of the risk of injuries to the user resulting from impact and/or abrasion. These factors are not sufficient to ensure ergonomics and comfort, due to the need to reach a consensus between weight, thermal load, and safety-related aspects. Designing protective products that take into account only material aspects does not allow determining the overall risk resulting from the loss of ergonomics or the introduced thermal discomfort [13,18,19,20]. Yousif et al. [21] also discusses aspects of motorcyclist visibility, often in the absence of it due to weather or climatic conditions, which is an important aspect of safety. The introduction of phase-change materials into textiles, which are the basis for the construction of clothing for motorcyclists, will increase the thermal comfort of users of this product [22,23].

Another aspect that should be taken into account in the design and verification of textile personal protective equipment is chemical safety. The content of chemicals in the fiber that may cause irritation and allergic reactions is definitely unacceptable from the point of view of the user’s health safety [24]. For this reason, it is important to carry out tests to verify quantitatively and qualitatively the content of substances causing the risk of allergic reaction and/or skin irritation.

The PN-EN 13595-1:2005 standard [13] also includes requirements for textile materials in the field of chemical safety, which boils down to the need to verify only the resistance to color change as an indicator indirectly indicating the risk of dye migration and, subsequently, the possibility of introducing a risk in terms of irritation, allergenic reaction, or, in extreme cases, carcinogenic effect. Such tests, in our opinion, are insufficient to reliably indicate the safety of a textile product and need to be extended to include other factors that migrate from the product to the user’s body and may cause health hazards.

The currently introduced series of EN 1621 standards [25,26,27,28] does not have a long-term response in the field of validation of test results with dangerous situations that occur in road traffic, especially in the redundant area in which police officers performing operational activities on motorcycles operate. For this reason, tests carried out in accordance with [13] seem to be more rational by choice, due to the extensive literature validating the methodologies contained in this standard.

The aim of the research was to effectively develop a textile solution to be used in the event of operational activities carried out by police officers—motorcyclists. The thesis of the research was the possibility of combining the principles and analyses of design thinking and usability engineering, which enable the development of an effective material and construction strategy in terms of ensuring performance and safety. The research also indicates the need to increase the scope of research in the area of chemical safety, which will allow for a full assessment of risks, including residual risks in relation to ensuring the safety of the user of the protective device.

The result of the above-mentioned activities was the verified prototype of a textile motorcycle suit for police officers carrying out operational activities in extreme situations and subjected to the risk of threats related to the possible consequences of road accidents (collisions, abrasions, damage to the textile barrier, etc.).

## 2. Materials and Methods

### 2.1. Materials

The following textile materials were used for the design of the motorcycle suits:

- Cordura 1100 woven fabric originated from C.F.WEBER GmbH (Leutersdorf, Germany); codded in Table 1 as C1100 layer;
- Cordura 560 woven fabric originated from C.F.WEBER GmbH (Leutersdorf, Germany); codded in Table 1 as C560 layer;
- Oratlion originated from YORK (Radom/Poland); codded in Table 1 as O layer;
- Black knitting mesh originated from GEDEON (Lodz, Poland); codded in Table 1 as BK layer;
- Waterproof membrane originated from Green Side (Warsaw, Poland); codded in Table 1 as WM layer;
- Oultlast lining originated from Outlast Technologies GmbH (Heidenheim, Germany); codded in Table 1 as OL layer;
- Navy blue mesh originated from GEDEON (Lodz, Poland); codded in Table 1 as NBK layer;
- Kevlar knit originated from INVENTEX (Hof/Saale, Germany); codded in Table 1 as KK layer;
- Black woven lining originated from C.F.WEBER GmbH (Leutersdorf, Germany); codded in Table 1 as BL layer;

The arrangement of material layers in individual Zones 1–3 of the designed motorcyclist’s suit defined in accordance with PN-EN 13595-1:2005 [13] is presented in Table 1.

The selection of materials and their configuration in the applied material system for individual risk zones in accordance with the PN-EN 13595-1:2005 standard [13] resulted from our previous experience and preliminary tests optimizing individual systems in order to meet the requirements of the above-mentioned standardization document. Table 1 presents the final effects of optimization of the configuration of individual material systems with matching, in accordance with the assumptions of the research for individual risk zones.

**Figure 1:**
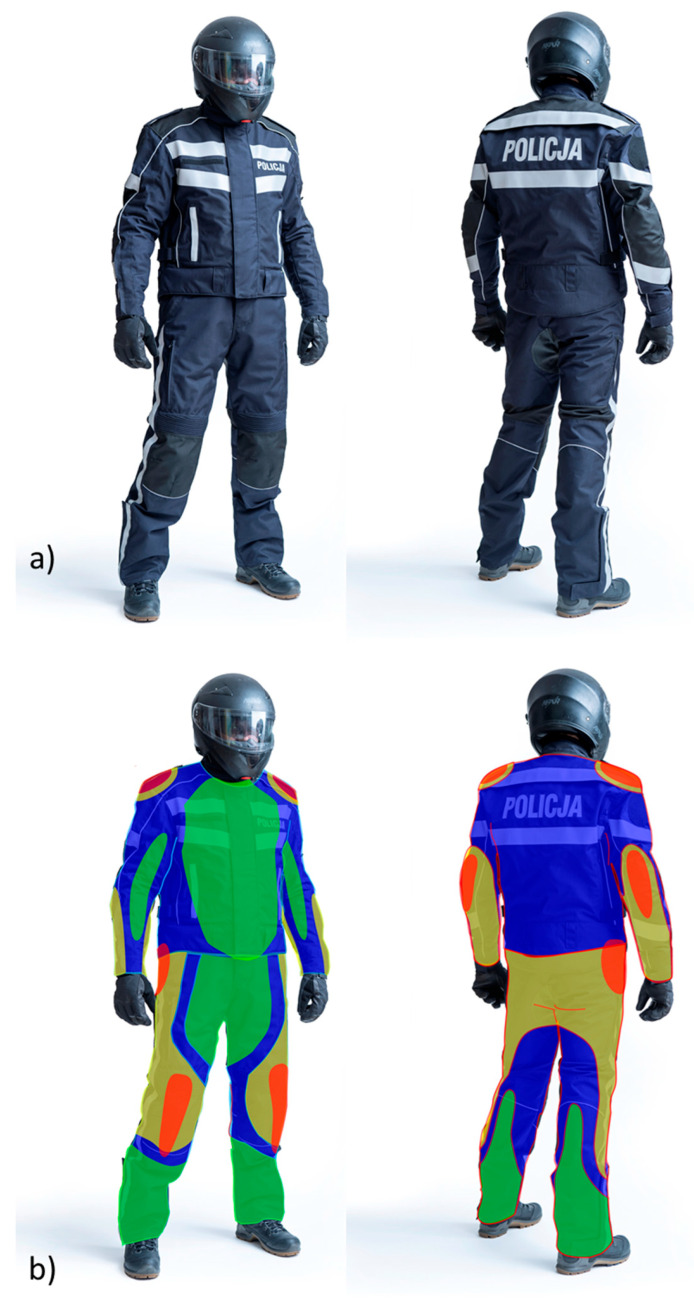
Prototype of the motorcycle suit (**a**) for policemen and the localization of Zone 1–3 (according to PN-EN ISO 13595-1:2005 [13]) on the designed prototype of the motorcycle suit for policemen (**b**): red—Zone 1 (impact protectors and high abrasion resistance); yellow—Zone 2 (high abrasion resistance); blue—Zone 3 (moderate abrasion resistance); green—Zone 4 (residual risk) [photography: Paweł Oborski; zone arrangement: Piotr Suchocki].

Identification of the localization of Zones 1–4 on the designed prototype of the motorcycle suit for policemen is shown in Figure 1.

The shoulders and elbows protectors (complied with PN-EN 1621-1:2013 standard [25]) as well as back protectors (complied with PN-EN 1621-2:2013 standard [26]) were originated from SAS-TEC GmbH (Markgröningen, Germany).

The prototype motorcyclist’s suit subjected to the verification process consisted of a summer jacket, a winter jacket, and trousers. The innovation of the above-described solution was described in [29,30,31,32].

### 2.2. Methods

#### 2.2.1. Design Thinking Methodology

Special-purpose clothing must be adapted to the needs resulting from the anatomy and physiology of the wearer, as well as to the purpose it is intended to serve. Such clarification allows for the use of optimally selected textile materials and innovative production methods, which increases the functionality of this garment. It is intended to serve special purposes, so it should meet specific technical and ergonomic requirements and parameters and not be subject to temporary fashions, which in turn are characteristic of haute couture clothing. The requirements for designing a new uniform that would meet the requirements of professional motorcyclist clothing had to be divided into two main pillars, i.e., functionality and aesthetics/appearance. The adopted style had to take into account the adaptation to the already existing uniform and be clearly recognizable and visible, as well as stand out significantly from other outfits of motorcyclists moving in public space. The second pillar is the requirements of the body (protection, anthropometry, ergonomics of movement, and thermophysical regulation) and the requirements of physical activity of the profession itself, i.e., driving a motorcycle and performing other business activities. Very important points of the roadmap were the discussion of initial designs with experienced users and the realization of these models and prototypes, which were then tested in real conditions.

#### 2.2.2. Usability Engineering

The aspects of the usability technology were adopted from the guide described in the PN-EN 62366-1:2015-07/A1:2021-03 standard [33].

#### 2.2.3. Estimation of the Safety of the Designed Motorcycle Suits

Dimensional change length

Dimensional change length was determined according to the procedure described in PN-EN ISO 4674-1:2017-02 standard [34].

Determination of the tear strength

The tear strength of the textile materials was determined according to the PN-EN ISO 4674-1:2017-02 standard [34].

Determination of the abrasion resistance

The abrasion resistance of the textile materials was determined according to the PN-EN 13595-1:2005 [13] and PN-EN 13595-2:2005 [35] standards.

Determination of the impact cut resistance

The impact cut resistance was determined according to the PN-EN 13595-1:2005 [13] and PN-EN 13595-4:2005 [36] standards.

Determination of the burst strength

The burst strengths were determined according to the PN-EN 13595-1:2005 [13] and PN-EN 13595-3:2005 [37] standards.

Determination of the pushing resistance

The pushing resistance was determined according to the PN-EN 13595-1:2005 [13] and PN-EN 13595-3:2005 [37] standards.

Conservation process

Changes in the tear strength, abrasion resistance, and impact cut resistance parameters were also verified prior to testing after 5 wash cycles in accordance with EN ISO 6330:2012 [38], procedure 3N, at a temperature of 30 °C.

The tests described in Section 2.2.3 were performed in the accredited SATRA Technology Centre Ltd. (Kettering, UK) [39,40,41].

#### 2.2.4. Chemical Safety

Amine content

Amine content in textiles was determined according to PN-EN 14362-1:2012 [42] and PN-EN 14362-3:2012 [43] standards.

Formaldehyde content

Formaldehyde content in textiles was determined according to the PN-EN ISO 14184-1:2011 standard [44].

pH of aqueous extract

The pH of aqueous extract from textiles was determined according to the PN-EN ISO 3071:2007 standard [45].

Heavy metal content in a mineralized sample of textiles was determined according to the PN-EN 16711-1:2016-01 standard [46], whereas in an extract from textiles, it was determined according to the PN-EN 16711-2:2016-01 standard [47].

Nickel content

Nickel content was determined according to the EN 12472:2005+A1:2009 standard [48].

## 3. Results and Discussion

### 3.1. Design Thinking

The entire design part, taking into account the aesthetics of design and ensuring the functionality of use, was carried out in several stages. The biggest difficulty turned out to be designing elements of the uniform that would meet aesthetic expectations. The external appearance had to be approved by both the police officers and the management of the Police Headquarters. Reconciling the interests and points of view of all parties, while taking into account the public perception of police uniforms in an unambiguous and desirable way, was a big challenge. In addition to the institutional requirements, the final non-verbal message was important, clearly indicating the uniformed service—the Police—and communicating the rank of representatives of this service. The need to meet all formal and informal requirements required many consultations, which in turn translated into a multi-stage design process and a relatively long time for implementation of individual projects. An additional challenge was functional adaptations in the aspect of two separate motor skills of the performed duties, i.e., both driving a motorcycle in a sitting position and carrying out inspections of drivers in a standing and walking position. The subject of considerations in this area was the types of adjustment and ways of adjusting to the sitting position, the layout and size of ventilation holes, the arrangement and types of reflective elements adapted to the protectors, the number and methods of tight fastening to protect against the wind, and the compatibility of the jacket with the pants. All these aspects determined the technical and construction solutions, meeting ergonomic, normative, quality, and functional requirements and improving safety and comfort of work. Work on designing professional clothing for special tasks also requires the cooperation of the designer with a team of co-creators, consisting of qualified and experienced specialists, among whom should be constructors, technologists, and material experts in textiles and additives used in the production of a given type of specialist clothing.

Choosing the style of a given assortment of clothing was also an important issue. The form, shape, and symbolism of the outfit related to history and accepted fashion canons give great opportunities for non-verbal messages evoking specific associations—at “first glance”, so the designer should take into account the system of visual signs that the outfit is a carrier. In this case, the external appearance is much more than just a specific aesthetic canon. It is a type of message whose content (and, of course, form) must be used responsibly, in accordance with the intentions of the end customer. Clothing, therefore, plays the role of a medium of non-verbal communication. The communication message must therefore be clear to everyone and unambiguous.

**Figure 2:**
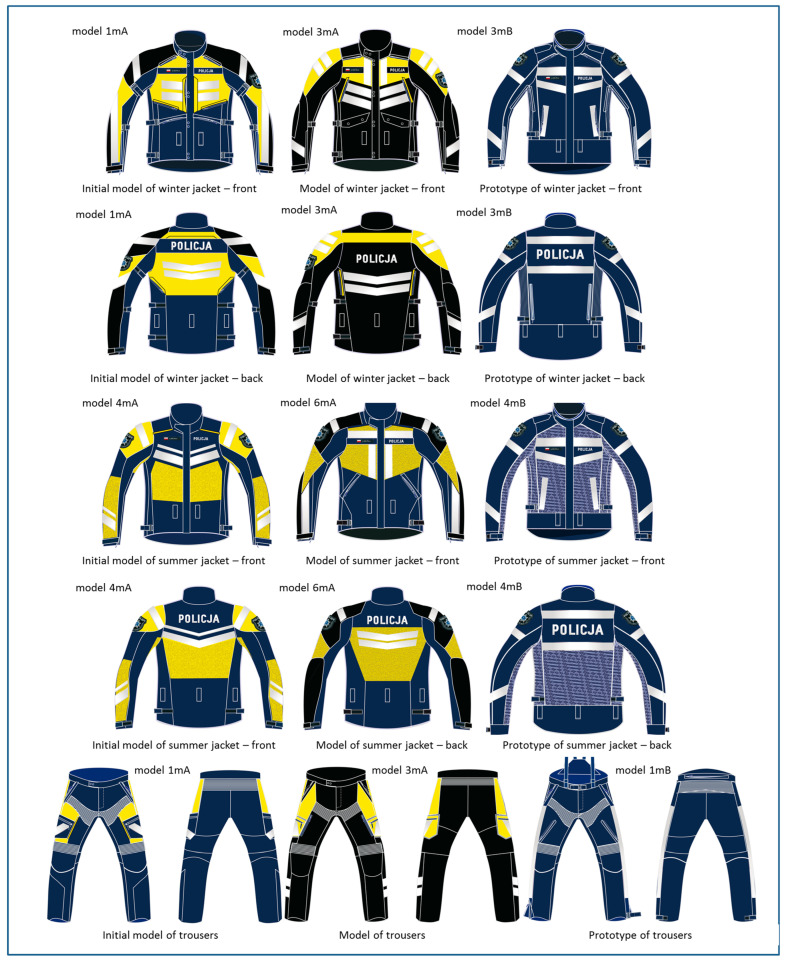
Examples of changes in the design of the winter as well as summer jackets and trousers of the motorcycle suit as a result of the performed design thinking process.

Figure 2 shows the changes resulting from the design thinking process implemented in the project in the visual communication on the example of selected models and a prototype of jackets (winter and summer) and trousers.

The use of all these solutions therefore includes both socio-psychological issues (recognition, arousing respect, respect, and trust) and the possibilities related to the properties of the material itself. Very important for the appearance was the selection of materials with reflective properties and increased color intensity, which gives a better result of emphasizing selected elements of the outfit and separating it from the “background”, which is the surroundings. This aspect seems to be important within urban agglomerations, as well as wherever attention is distracted by light elements, for example, illuminated banners, displayed advertisements, or brightly colored signboards that distract drivers. All these “forms of artificial attention” are as intriguing as they are dangerous, especially when they occur in places of increased vehicle and pedestrian traffic. Reflective tapes have been incorporated so that the motorcyclist-policeman stands out from the group of other motorcyclists and is visible to car drivers on public roads. This layout had to meet the required standards of protective clothing for professional motorcyclists (PN-EN 13595-1:2005 standard), which in particular determined the location of pockets for protectors—elements (material systems) protecting against falls.

All this had to be balanced with the conditions of textile production—such as the order of sewing the elements, joining elements of different materials, gluing reflective elements using the thermal transfer method, etc. The suit is made mostly of high-strength Cordura^®^ woven fabric, with SAS-TEC protectors (Markgröningen, Germany), confirmed by EN 1621-1:2012 [25] and reflective elements by 3M Scotchlite Reflective Material.

During the design research, 6 models of individual elements of the motorcyclist-policeman’s suit were developed, differing in the design and arrangement of external elements, resulting in the generation of non-verbal messages. These variants were verified by direct users, which was the basis for selecting the final aesthetics of the prototype.

A significant challenge in creating new solutions in the design of the suit elements was to maintain a clean and consistent form, in line with the adopted concept of an original appearance that stands out from other users of two-wheelers, while placing all solutions and elements supporting motorcycle clothing and maintaining a consistent appearance with the already existing uniforms. In the opinion of the motorcyclist police officers delegated to cooperate, the final demonstrator is functional and aesthetically consistent, and its individual modules are refined and compatible. The aesthetics refer to the current uniform and meet the criterion of original and clearly visible special clothing for the police.

### 3.2. Usability Engineering

Usability engineering is the basic element of the process of analysis and usability assessment of the designed product, directly referring to its safety. This process allowed us to assess and minimize the risks associated with the correct use and errors of using the policeman-motorcyclist’s suit.

As part of the research, a sequence of events was defined which, performed by the user of the device, will increase the risk of its use and/or generate new risks.

Scenarios for the use of the textile motorcycle suits related to the most important aspects have also been defined for:

- every day, typical use;
- redundant use;
- use in extreme conditions (e.g., at temperatures above +50 °C or below −30 °C);
- inconsistent with the maintenance process indicated by the manufacturer;
- illegible or overly complicated instructions for use;
- illegible label;
- too long time between trainings;
- insufficient quality of training;
- improper primary/secondary packaging;
- incompatible storage/transport conditions of the textile motorcycle suits;
- lack of checking the skills of the direct user;
- accidental damage to the product during use and the procedure for reporting/replacing the textile motorcycle suits;
- use of the textile motorcycle suits after the defined period of use;
- incorrect drying process of the product after use in rain/snow;
- failure to provide the comfort required by the specificity of use, including thermal comfort and ergonomics;
- quick wear of the materials and protectors used;
- inappropriate size design;
- too high exposure to a collision;
- too high exposure to abrasion;
- exposure to carcinogenic, skin-irritating, and sensitizing substances (substance migration from the man-made fibers);
- loss of safety after maintenance processes.

This allowed us to generate a list of issues related to the use of the designed textile motorcycle suits, which were introduced to the application test survey—the process of validation of the designed suit in real conditions (which is another aspect of the research work).

The results of the above-mentioned processes have been included in the form of records in the training documentation and instructions for use of the product, as well as on the label. In this article, attention will be focused on aspects related to ensuring the safety of the user, taking into account the risks resulting from road incidents, often specific and resulting from the actions carried out by police officers. It takes into account the risk of hard injuries, abrasions, and the impact of sharp objects on soft tissues.

### 3.3. Safety Assessment

The designed policeman-motorcyclist suit consists of 3 basic textile parts: trousers, a summer jacket, and a winter jacket. The process of verification of the safety of the prototype product was carried out in accordance with the results of analyses carried out as part of the design thinking process and usability engineering, as well as records and guidelines, in particular normative documents from the PN-EN 13595 standards series [13,35,36,37].

**Figure 3:**
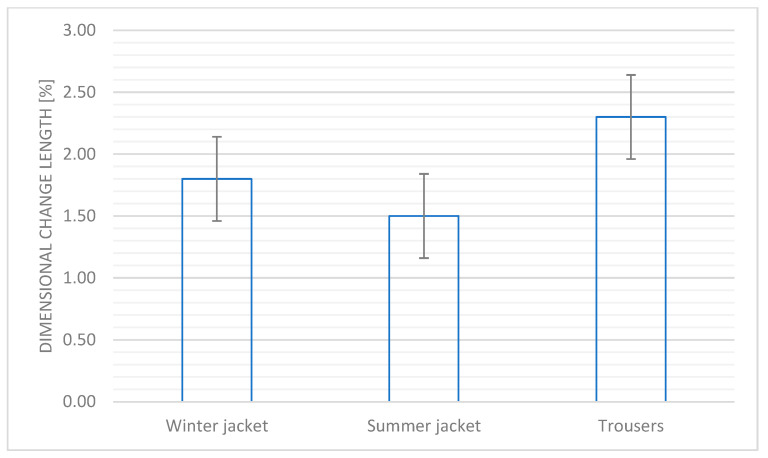
Dimensional change in length of the textile systems after 5 washing cycles estimated for the winter and summer jackets and for trousers.

Figure 3 shows the changes in dimensional change length after 5 washing cycles evaluated for each part of the system: the winter and summer jackets and the trousers.

The requirement for the designed textile product in accordance with the PN-EN 13595-1:2005 standard [13] is not to exceed the linear dimensions of the material from individual elements of the suit by more than 3%.

The changes observed for individual elements of the prototype clothing did not exceed the required value of this parameter for both trousers, summer jacket, and winter jacket, which allows us to confirm the dimensional durability of the prototype protective clothing.

The system of dividing the area of protective clothing into zones results from the assessment of the risk resulting from accidents related to collisions, abrasion, cutting, and other mechanical damage resulting in the loss of continuity of textile protection. For the above reasons, four risk zones have been adopted in accordance with the PN-EN 13595-1:2005 standard [13]: Zone 1 with a high risk of impact, Zone 2 with a high risk of abrasion, Zone 3 with a moderate risk of abrasion, and Zone 4 with a slight risk of abrasion. In the case of the solution that is the subject of this research, three danger zones have been defined: Zones 1–3.

**Figure 4:**
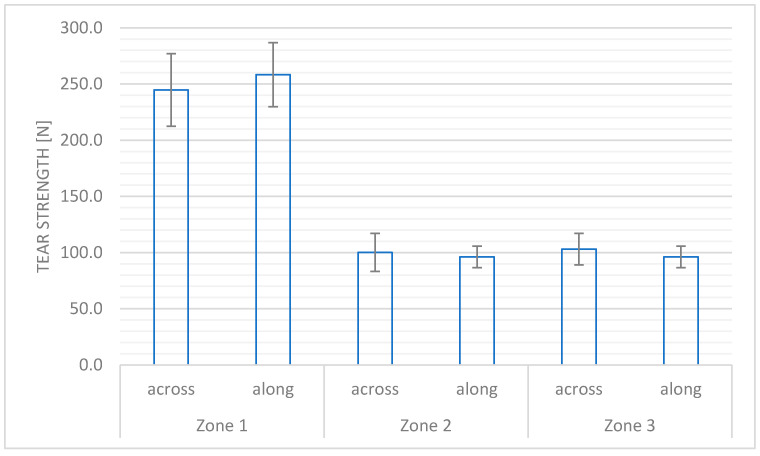
The tear strength for the zones defined for the designed motorcycle suit.

The tear strength of the material system from each zone defined for each part of the motorcycle suits is presented in Figure 4.

In accordance with the requirements of PN-EN 13595-1:2005 [13], the tear strength parameter of the main textile material for all zones specified in the product should not be lower than 70 N. For the C1100 woven fabric used in Zone 1, the value was over 3.5 times higher than required, which indicates high safety of the product in the context of collision and abrasion. The test results for Zone 2, where the main component is C560 woven fabric, showed the tear strength was 1.5 times higher than the level of this parameter indicated as the minimum in the PN-EN 13595-1:2005 standard [13]. Similar values were obtained for the textile component used in the least risk zone.

**Figure 5:**
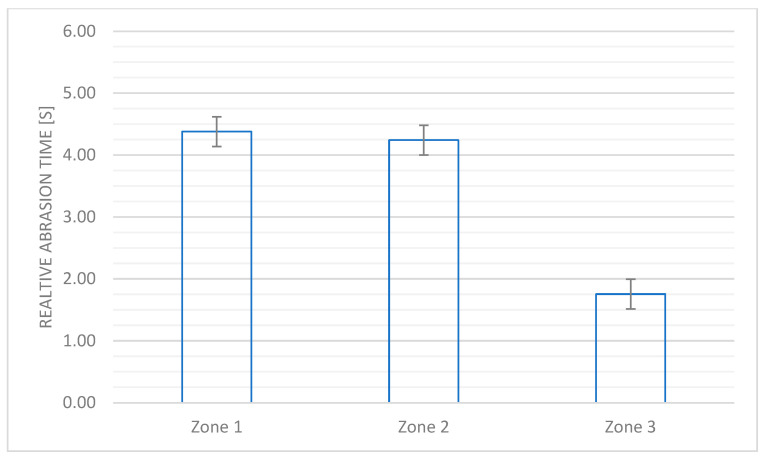
The abrasion resistance for Zone 1, 2, or 3 of the designed motorcycle suit according to the PN-EN 13595-1:2005 standard [13].

The values of the abrasion resistance determined for each zone of the prototype of the motorcycle suit were presented in Figure 5.

In the case of Zones 1 and 2, where there is a significant risk of abrasion of the textile barrier, the minimum value of the abrasion time for Level 1 should not be less than 4.0 s. In the case of Zone 3, the value of this parameter at Level 1 should not be lower than 1.8 s. All textile systems used in the protective product type met the requirements defined for individual protective devices, which confirmed the user’s safety against injuries related to the abrasion of the textile barrier and possible mechanical injuries to the body.

**Figure 6:**
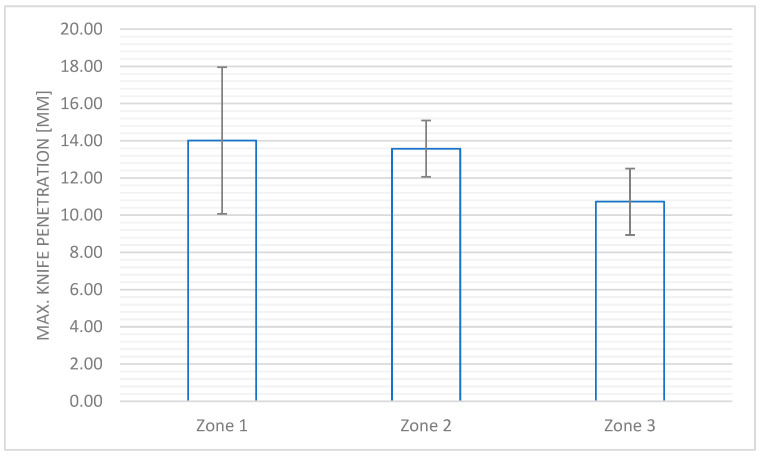
The impact cut resistance for Zone 1, 2, or 3 of the designed motorcycle suit according to the PN-EN 13595-1:2005 standard [13].

Figure 6 shows the impact cut resistance determined by the maximal value of the knife penetration into the textile systems applied in Zones 1–3.

During the examination of individual zones in the motorcyclist’s clothing in terms of resistance to impact cutting, the normative requirements specified for Zones 1–2 (knife penetration velocity of 2.8 m/s) and Zone 3 (knife penetration velocity of 2.0 m/s) were taken into account. For all zones of the developed suit, average results of maximum knife penetration were obtained, classifying them to Level 2 (the requirement for the limit of the knife penetration depth in the case of Zones 1–2 should be lower than 15 mm, and in the case of Zone 3—not higher than 25 mm), which confirms the high resistance of the textile systems used to penetration by sharp objects. The designed textile barrier is important for the user’s safety in terms of hazards resulting from the penetration of tools and sharp objects through it.

**Figure 7:**
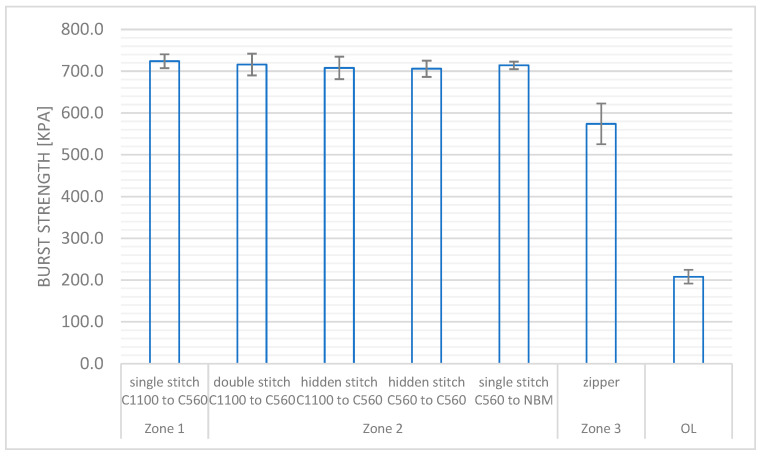
The burst strength determined for Zone 1, 2, or 3 and the lining of the designed motorcycle suit according to the PN-EN 13595-1:2005 standard [13].

The values of burst strength of the stitches applied in each zone as well as determined for the lining were shown in Figure 7.

The minimum values of the ejection strength specified for Zones 1 and 2 are not less than 700 kPa, while for Zone 3 not less than 500 kPa, and for the lining not less than 200 kPa. In the case of the material system applied in Zone 1 (variations of C1100 and C560 stitches incl.: hidden, single, or double stitches), the average value of this parameter is significantly higher than the Level 1 required by the standard.

Similarly, for the material systems used in Zone 2, average levels of this parameter above the required level were obtained. The zipper used in the suit also met the requirements for push-out resistance defined for Zone 3. The average value of burst strength measured for the OL lining was 208 kPa, being higher than the minimum set in the PN-EN 13595-1:2005 standard [13].

### 3.4. Chemical Safety Aspect

The chemical safety of protective textile products used in redundant conditions is an important aspect determining the fulfillment of the requirement to ensure full safety of the product user.

All textile materials used in the design of the prototype of the motorcyclist’s suit were characterized by the amine content significantly below the acceptable value of 30 mg/kg. A similar situation was observed in the case of formaldehyde content (<75 mg/kg), the content of heavy metals (in the mineralized sample the cadmium values were below 40.0 mg/kg for each tested material), and the content of arsenic and mercury in the sample of extract from individual textile materials, which achieved values of <1.0 mg/kg and <0.02 mg/kg, respectively. Moreover, the nickel content was also not exceeded (<0.05 μg/cm^2^).

Taking into account the above observations, it should be concluded that the residual risk present in the product in terms of the risk of skin irritation, allergenicity, and carcinogenicity is at an acceptable level.

## 4. Conclusions

The combination of design thinking techniques, usability engineering, and material strategy taking into account current normative requirements is conducive to the design of a textile product that ensures safety and the possibility of effective implementation, especially in the case of products directed to special areas of application and operational activities. Developed as part of interdisciplinary scientific research and development work, the motorcyclist-policeman suit provides, thanks to interaction with users, building designers’ awareness of the real needs of users and skillful creation of the material and construction strategy of the designed protective clothing. The prototype of the motorcyclist-policeman suit has been tested in a wide range of requirements, shaping knowledge about its safety in excessive use. The scope of normative tests focused on the PN-EN 13595-1:2005 standard [13] allowed us to learn about its ability to ensure safety in a wide range of potentially threatening situations, along with the definition of worst-case scenarios that may generate these events with a significant risk to life, health, and the user. The textile product designed in this way has proven its safety in terms of tear strength, abrasion resistance for Level 1 (Zones 1–3), impact cut resistance, and bursting strength for Level 1 (Zones 1–3). Chemical safety tests, carried out in a much wider scope than proposed by the PN-EN 13595-1:2005 standard [13], have shown no risk of migration of substances and metals potentially generating the risk of irritation, allergenicity, and/or carcinogenicity. From our point of view, tests designed in this way for the migration of chemical substances and heavy metals important for the safety allow us to determine the acceptability of residual risks related to the possibility of skin irritation, allergenic reaction, and, in the long term, neoplastic lesions (during long-term exposure).

The next stage of the research will be the evaluation of the product as part of validation carried out in real operating conditions carried out by police officers—motorcyclists during four seasons.

## Figures and Tables

**Table 1:** Characteristics of the material systems of the prototype motorcyclist’s suit in individual Zones 1–3 in accordance with the PN-EN 13595-1:2005 standard [13].

Zone acc. PN-EN 13595-1:2005 Standard	Material Arrangement in Material System (Top Layer → … → Bottom Layer)
1	C1100 → O → O → OL → WM → BK
2	C560 → KK → OL → WM → BL
3 ^1)^	C560 → OL → WM → BK

^1)^—in the designed solution, the material system that was tested for compliance with the required requirements described for zone 3 was also used in zone 4 in accordance with the PN-EN 13595-1:2005 standard [13]. For this reason, tests were conducted to confirm compliance with the parameters defined for the higher requirements of Zone 3. In addition, it should be noted that Zone 4 is treated as an area for which risks are acceptable from the point of view of potential exposure at the time of a hazardous event.

## Data Availability

The raw data supporting the conclusions of this article will be made available by the authors on request due to privacy/ethical.
